# Impact of exercise type, duration, and intensity on depressive symptoms in older adults: a systematic review and meta-analysis

**DOI:** 10.3389/fpsyg.2024.1484172

**Published:** 2024-09-13

**Authors:** Xinglu Li, Shaokai He, Tao Liu, Xinxin Zhang, Wenfei Zhu, Chao Wang, Yuliang Sun

**Affiliations:** ^1^School of Physical Education, Shaanxi Normal University, Xi’an, China; ^2^Fuzhou Preschool Education College, Fuzhou, China; ^3^School of Physical Education, Guangxi Normal University, Guilin, China

**Keywords:** physical exercise intensity, physical exercise type, depressive symptoms, older adults, meta-analysis

## Abstract

**Objective:**

This systematic review and meta-analysis assessed the effects of three types of physical exercise (resistance exercise, aerobic exercise, and group exercise), different exercise intervention times (3 months, 6 months), and different exercise intensities (low, moderate, and high) on the improvement of depressive symptoms in older adults aged ≥60 years, as well as to explore the impact of the sustainability of these physical exercise intervention programs on depressive symptoms in older adults.

**Methods:**

The randomized controlled trials (RCTs) on the effect of physical exercise on depressive symptoms in older adults were retrieved from Cochrane Library, Web of Science, PubMed, and Embase Data. The retrieval time limit is from establishing the database to January 7, 2024. We conducted a meta-analysis using a 95% confidence interval (95% CI) and the standardized mean differences (SMD). The *I^2^* statistic was used to assess the heterogeneity of the outcomes of the studies. When *I*^2^ < 50%, we used the fixed-effects model, and when *I*^2^ > 50%, we used the random-effects model. Subgroup and sensitivity analyses investigated heterogeneity origins.

**Results:**

There are 15 articles reported 20 studies, with a total of 1,346 patients, including 689 in the control group and 657 in the experimental group. The findings demonstrated a notable improvement in depression symptoms among older persons as an immediate result of engaging in physical exercise [SMD = −0.82, 95% CI (−1.19, −0.45)]. The subgroup analysis showed that moderate-intensity physical exercise [SMD = −0.25, 95% CI (−0.47, −0.03)], high-intensity physical exercise [SMD = −0.94, 95% CI (−1.37, −0.51)], resistance exercise [SMD = −0.70, 95% CI (−1.20, −0.20)], and group exercise [SMD = −0.97, 95% CI (−1.89, −0.05)], and the exercise intervention time was 3 months [SMD = −0.81, 95% CI (−1.38, −0.23)] or 6 months [SMD = −0.93, 95% CI (−1.46, −0.41)] were more effective in improving depressive symptoms in older adults.

**Conclusion:**

The sustainable resistance and group exercise have a better effect on improving depressive symptoms in older adults. Appropriate exercise intervention time can also ensure the sustainable improvement effect of exercise.

**Systematic review registration:**

https://www.crd.york.ac.uk/PROSPERO/record_email.php, identifier CRD42023405525.

## 1 Introduction

Depression is a common condition which impacts about 3.8% of the world’s people, including 5.0% of adults and 5.7% of those aged 60 and above (WHO, 2023). The World Health Organization estimated that depression will be a significant burden on global health by 2030 (Dwyer et al., 2020), coinciding with a expected increase in the population of older adults aged 60 and above from 1 billion to 1.4 billion (World Health Organization, n.d.). With China entering an aging society, there has been a rise in the number of older adults living alone, resulting in geriatric depression emerging as a public health concern. The survey data indicates that 33.1% of older adults aged 60 and above in China exhibit depressive symptoms. Older depression is defined by its high occurrence, heightened suicide rates, and low recognition and consultation rates, and its management is challenging due to poor tolerance of pharmacotherapy and its frequent association with chronic illness.

Because of the prevalence and cost of treatment associated with mental health disorders, exercise has emerged as a viable subsidiary or alternative strategy in the treatment process (Pedersen and Saltin, 2015). There is a widespread belief that physical exercise can positively impact moods, anxiety, and overall physical and psychological well-being, leading to its widespread recommendation for treating depression (Stanton and Reaburn, 2014; Ravindran et al., 2018). Some studies have shown that physical exercise helps to alleviate mild to moderate depression, anxiety, and panic disorder (Brosse et al., 2002). A recent study review also suggests physical exercise positively affects depression (Cooney et al., 2013). In addition, exercise may improve perceived functioning and reduce social burden (cost of social support) in older adults (Fox et al., 2007). Although there are many studies on the effects of physical exercise on depressive symptoms in older adults, different studies have used various indicators to reflect depressive symptoms in older adults. Most of the interventions studied used aerobic exercise that can improve cardiovascular function, such as walking (Pedersen and Saltin, 2015; Mikkelsen et al., 2017). Other studies have also suggested that resistance training and related training ways are more effective in improving depressive symptoms in older adults than aerobic exercise (Cooney et al., 2013). Secondly, the experimental methods varied widely across studies, such as exercise vs. placebo, exercise vs. medication, exercise vs. no other intervention, and exercise vs. psychotherapy. In addition to this, the experimental settings varied across studies, such as environment (indoor exercise vs. outdoor exercise), social contact (group exercise vs. individual exercise), and autonomy (supervised exercise vs. unsupervised exercise) (Pedersen and Saltin, 2006).

Therefore, this study aims to systematically assess the existing study on the impact of physical exercise on depressive symptoms in older adults. It seeks to include a wider range of outcome indicators to comprehensively examine the effects of aerobic, resistance, and group exercise on alleviating depressive symptoms in older adults. The findings of this study aim to offer a theoretical foundation for selecting suitable exercise modalities for older adults, and at the same time provide valuable insights for the development of effective and sustainable intervention strategies, which can enhance quality of life and improve well-being in older population.

## 2 Materials and methods

This study’s implementation process and report writing strictly used the PRISMA (Preferred Reporting Items for Systematic Reviews and Meta-Analyses) standards as a guide, ensuring methodological rigor and comprehensive reporting. The Cochrane Handbook for Systematic Reviews of Interventions was also adhered to, further enhancing the quality and reliability of the methodology (Cochrane Training, n.d.). Additionally, this study was registered with PROSPERO (International prospective register of systematic reviews) under the registration number CRD42023405525, reinforcing transparency and adherence to established protocols in systematic reviews.

### 2.1 Search strategy

This study thoughly searched the published studies examining the relationship between physical exercise and depressive symptoms in older adults in several prominent databases, including the Cochrane Library, Web of Science, PubMed, Embase, CNKI, and Wanfang. The search included studies available until 7 January 2024. We have adopted a comprehensive search strategy to maximize the scope of the search with key terms. The english search terms employed were “exercise,” “older adults “, and “depression,” while the chinese search terms used were “physical exercise,” “older adults “, and “depression.” In addition, the search extended to grey literature sources to capture unpublished or non-peer-reviewed studies, although no relevant results were found in these sources. We also reviewed the references of included studies to ensure that all pertinent literature was considered.

### 2.2 Eligibility criteria

Inclusion criteria: (1) The research design must be randomized controlled trials (RCTs); (2) The participants must be healthy older adults aged over 60 years; (3) Before the exercise intervention, there should not be any significant differences between the experimental group and the control group; (4) The experimental intervention must involve physical exercise, while the control group should not take part in any regular exercise (CT); (5) The outcome indicators should include the Geriatric Depression Scale (GDS), Symptom Checklist 90 (SCL-90), Geriatric Depression Scale-15 (GDS-15), the Korean version of the Geriatric Depression Scale (SGDS-K), the 18-item Taiwanese Depression Questionnaire (TDQ) and the Thai Geriatric Depression Scale (TGDS); (6) The research data should be reported in the form of mean ± standard deviation (M ± SD); (7) The articles in either Chinese or English languages were considered for inclusion.

Exclusion criteria: (1) Older adults with comorbidities or below 60 years of age; (2) Articles includes interventions beyond exercise; (3) Studies with incomplete data or lacking a control group.

### 2.3 Data extraction

Based on the specified criteria for inclusion and exclusion, the data extraction from the included studies was performed by two authors, who resolved any disagreements through negotiation or consultation with a third author. The extracted information from the studies included various aspects, including research characteristics (year, country, author), subject characteristics (age, sample size), intervention details (measures, intervention time, frequency), and outcome indicators. Moreover, when a single article contained multiple studies, the data were extracted individually. One author was in charge of getting the data, and the other was in charge of making sure the data was correct. In situations where the data was missing, we endeavored to get in touch with the author to obtain the necessary information.

### 2.4 Risk of bias assessment

The Cochrane bias risk assessment tool was employed, comprising six components: selection (including random sequence generation and allocation concealment), implementation (including blinding of investigators and participants), measurement (blinding of study outcomes), follow-up (completeness of outcome data), reporting (selective reporting of study results), and other (potential sources of bias). Each project underwent evaluation, categorized into three levels: low, unclear, and high risk of bias. The risk of bias assessment was performed using RevMan, and a bias risk map was generated. Sensitivity analysis was conducted using Stata software. Two authors independently assessed the risk of bias using the Cochrane Risk of Bias (RoB) tool (Sterne et al., 2019), and any differences were addressed by consensus or consultation with a third author.

### 2.5 Statistic methods

For this meta-analysis, the Review Manager 5.3 software and Stata 16.0 software were utilized. As all outcome data of the included studies were continuous variables, the standardized mean difference (SMD) and 95% confidence intervals (95%CI) were used as effect sizes for the analysis. The study heterogeneity was assessed using the *I*^2^ statistic. If the heterogeneity test did not indicate statistical significance (*I*^2^ < 50%; *p* > 0.05), the fixed-effects model was applied. Conversely, a random-effects model was employed when significant heterogeneity was observed (Zhou et al., 2023). Furthermore, the intensity of physical exercise is classified according to the “Chinese Society of Sports Science Group Standard T/CSSS 002–2023 (National Group Standards Information Platform, n.d.), the subgroup and sensitivity analyses were used to investigate potential sources of heterogeneity. A level of *p* < 0.05 was deemed statistically significant.The study’s publication bias was evaluated using Egger’s test and funnel plots (Aymerich et al., 2024). The Egger test is used to detect asymmetry in the funnel plot, with a *p*-value threshold of <0.10 indicating potential publication bias (Egger et al., 1997).

## 3 Results

### 3.1 Study selection

**Figure 1:**
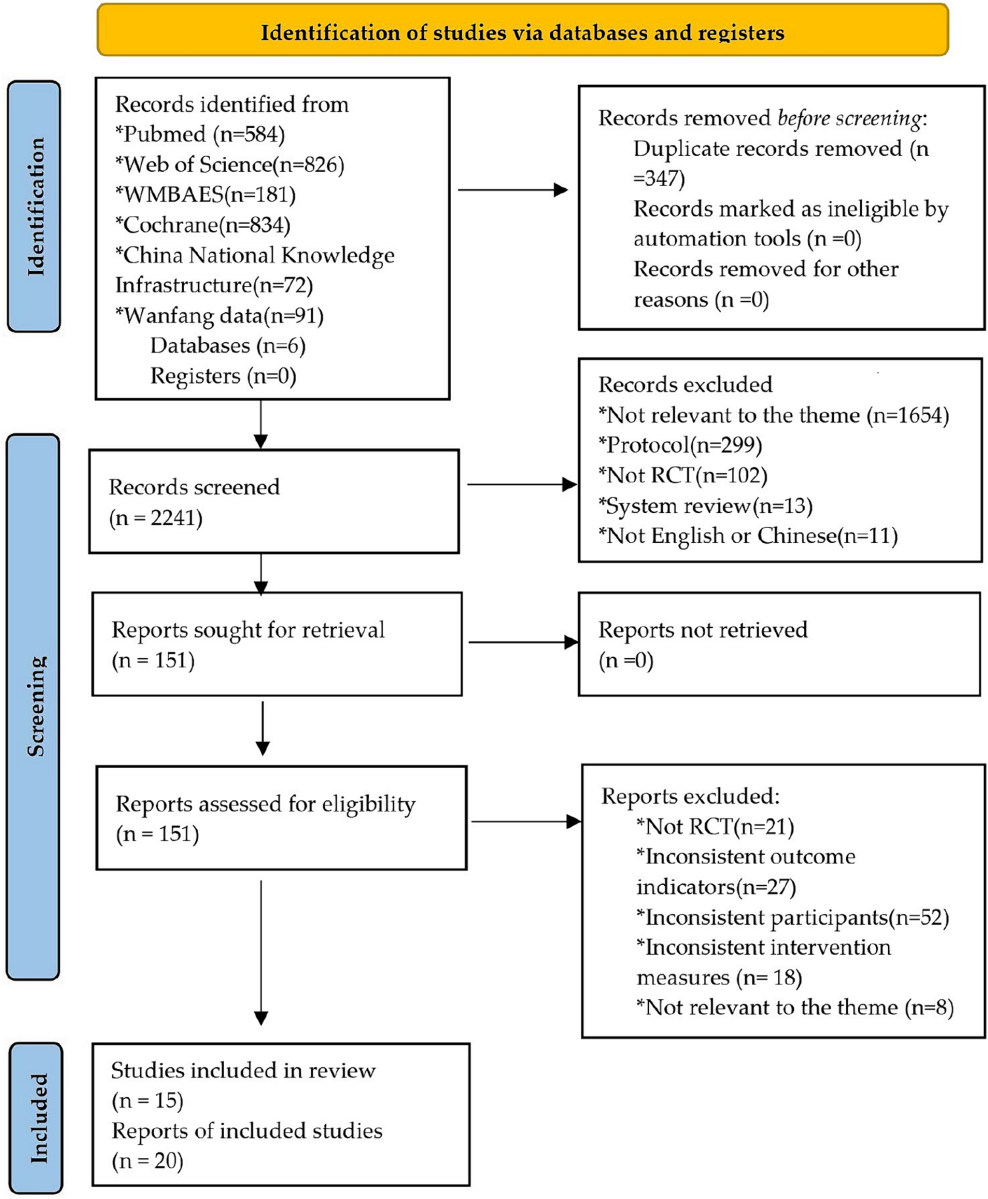
PRISMA flowchart of the study selection process.

Figure 1 displays the article screening process, which is shown as a flow chart. It provides an overview of the study selection process and the reasons for excluding research. Based on the Mesh and synonyms published up until January 7, 2024, we have discovered a total of 2,588 research studies from the databases. After removing 347 duplicate instances, a total of 2,241 studies remained. After reviewing the title and abstract, 1,336 papers were excluded. Based on the remaining 151 studies, we removed 131 after conducting a thorough examination of the whole text, based on the specified criteria for inclusion and exclusion. The final sample included 20 studies, including 4 Chinese articles (Ma et al., 2012; Lyu, 2012; Guan, 2017; An, 2019) 11 English articles (Shahidi et al., 2011; Antunes et al., 2005; Huang et al., 2015; Kim et al., 2019, 2021; Apóstolo et al., 2019; Laredo-Aguilera et al., 2018; Cunha et al., 2022; Makizako et al., 2019; Chen et al., 2015; Prakhinkit et al., 2014). The article screening process is shown in Figure 1.

### 3.2 Study characteristics and quality evaluation

There are articles involved 1,487 participants, including 758 in the control group and 729 in the experimental group. Three articles provided data for three comparisons (Guan, 2017; Shahidi et al., 2011; Makizako et al., 2019), and one provided data for three comparisons (Lyu, 2012). Therefore, 13 articles reported 18 studies. Three articles reported GDS scale scores; two reported SGDS-K scale scores; four reported GDS-15 scale scores, four reported SCL-90 scale scores, one reported TGDS scale scores and one reported TDQ scale scores. The study characteristics are shown in Table 1. The study quality and risk assessment are shown in Supplementary Figures S1, S2.

**Table 1:** Study characteristics.

Research	Region	Sample size	Age	Experimental group	Period	Frequency	Control group	Outcome indicators
Antunes et al. (2005)	Brazil	46	60+	Aerobic fitness training	6 months	3 times a week, on the other day	NE	GDS
Shahidi et al. (2011)	Iran	70	65+	Yoga, Aerobic	/	10 Times	NE	GDS
Huang et al. (2015)	Taiwan	57	65+	Physical training	12 weeks	3 times a week	NE	GDS
Laredo-Aguilera et al. (2018)	Spain	38	65+	Functional training (FT)	10 weeks	3 times a week for 60 min	NE	GDS-15
Apóstolo et al. (2019)	Portugal	44	70+	Physical exercise	12 weeks	3 times a week	NE	GDS-15
Kim et al. (2019)	Korea	21	65+	Strength training	24 weeks	3 times a week	NE	SGDS-K
Makizako et al. (2019)	Japan	89	65+	Exercise intervention, Horticultural activities	20 weeks	3 times per week for 30 min	NE	GDS-15
Kim et al. (2021)	Korea	224	65+	Walking, Gymnastics	7 months	weekly	NE	SGDS-K
Cunha et al. (2022)	Brazil	41	60+	Resistance exercise	12 weeks	3 times a week	NE	GDS-15
An (2019)	Chongqing	180	66+	Physical exercise	1 year	/	NE	SCL-90
Guan (2017)	Henan	48	60–70	Square Dance, Wuqinxi	3 months	3–4 times per week for more than 50 min	NE	SCL-90
Lyu (2012)	Shaanxi	209	60–70	Tai Chi, Square dance, and Ballroom dance	12 weeks	Greater than 30 min each time three times a week	NE	SCL-90
Ma et al. (2012)	Anhui	120	60–70	Sport walking	4 months	4 to 6 times/w, 120 min/time, 120 steps/min	NE	SCL-90
Prakhinkit et al. (2014)	Thailand	45	60–90	Walking	12 weeks	3 times a week	NE	TGDS
Chen et al. (2015)	Taiwan	114	75–84	Elastic band	6 month	3 times a week	NE	TDQ

NE, No physical exercise; GDS, Geriatric Depression Scale; SCL-90m, Symptom Checklist 90; GDS-15, Geriatric Depression Scale-15; SGDS-K, Korean version of the Geriatric Depression Scale; TGDS, Thai Geriatric Depression Scale; TDQ:18-item Taiwanese Depression Questionnaire.

### 3.3 Results of a meta-analysis

**Figure 2:**
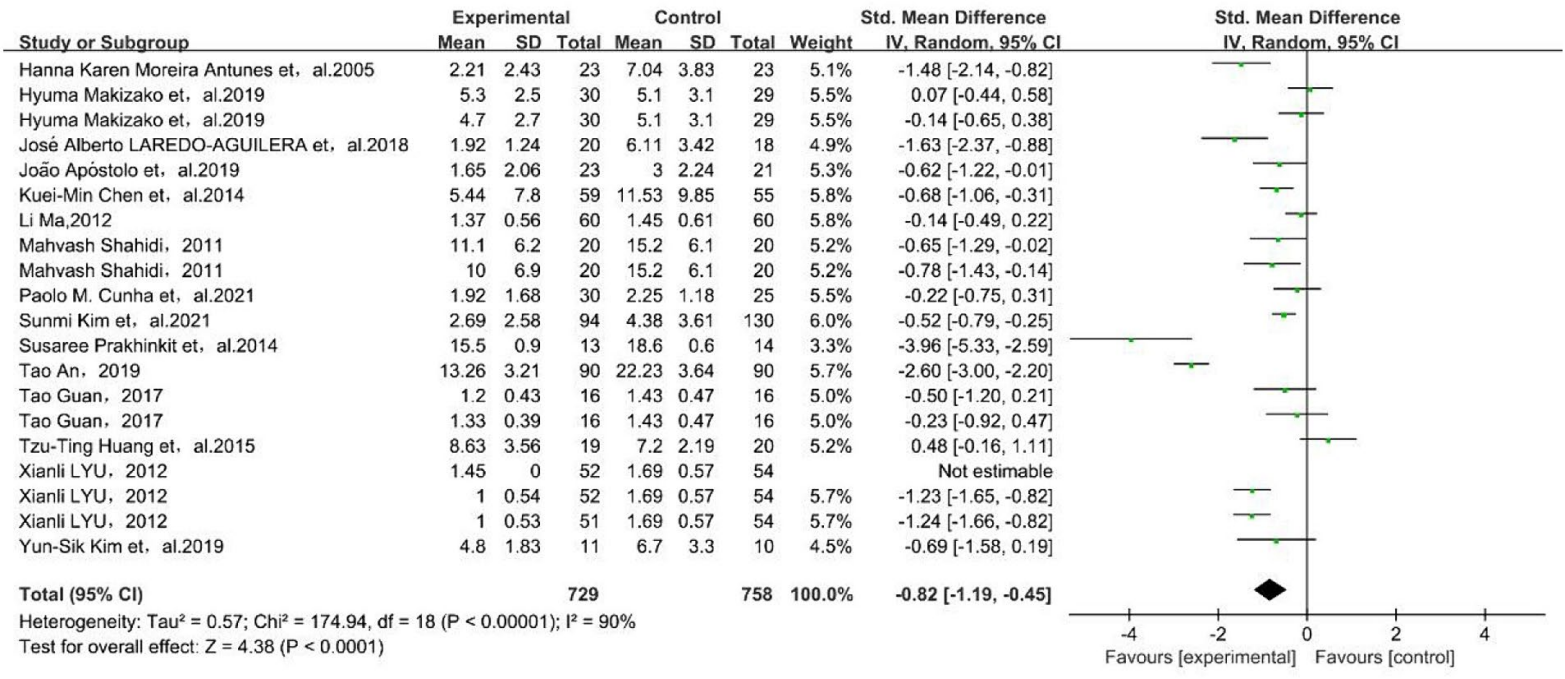
Forest plot for meta-analysis of physical exercise on depression in older adults. The green box and the black rhombus represent results of the individual studies and the combined results, respectively.

A total of 18 studies used the Depression Scale as an outcome indicator and reported the effect of physical exercise on depressive symptoms in older adults. The 18 studies were tested for heterogeneity (*I*^2^ = 90%, *p* < 0.01), and a random-effects model was used to combine the results. The results showed that the experimental group helped older adults with depression more than the control group [SMD = −0.82, 95% CI (−1.19, −0.45)], as shown in Figure 2.

To explore the sources of heterogeneity, the subgroup analyses were conducted on factors that may cause heterogeneity, with exercise intervention time, physical exercise intensity, and exercise type as subgroup variables. The results showed a decrease in heterogeneity between studies, suggesting that exercise intervention time, physical exercise intensity, and exercise type may be sources of heterogeneity.

#### 3.3.1 Results of the exercise time subgroup analysis

**Figure 3:**
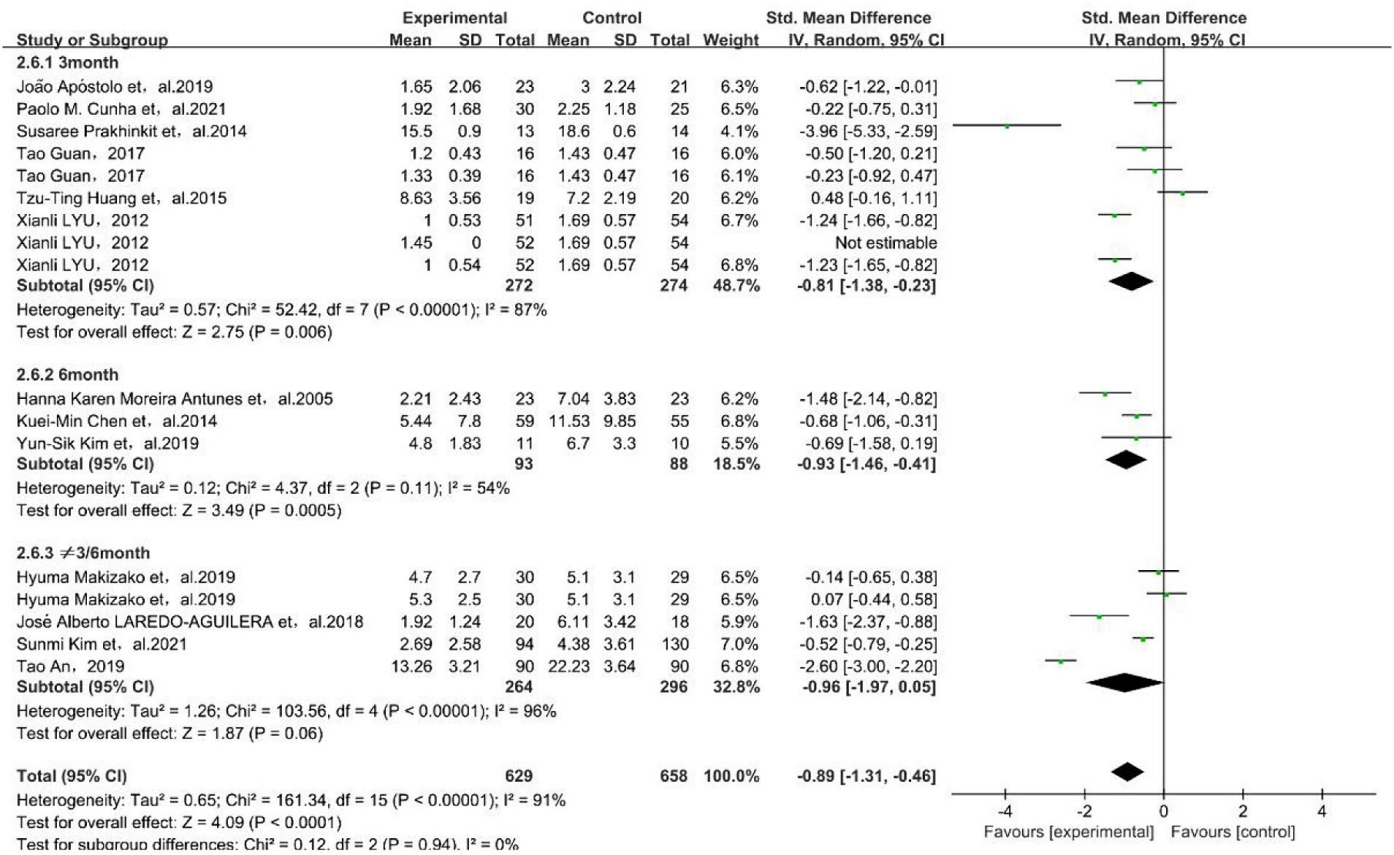
Forest plot of subgroup meta-analysis of intervention time. The green box and the black rhombus represent results of the individual studies and the combined results, respectively.

Both the three-month [SMD = −0.81, 95% CI (−1.38, −0.23)] and six-month [SMD = −0.93, 95% CI (−1.46, −0.41)] exercise intervention time had a significant improvement in depressive symptoms in older adults, and 6 months was better than 3 months, as shown in Figure 3.

#### 3.3.2 Results of physical exercise intensity subgroup analysis

**Figure 4:**
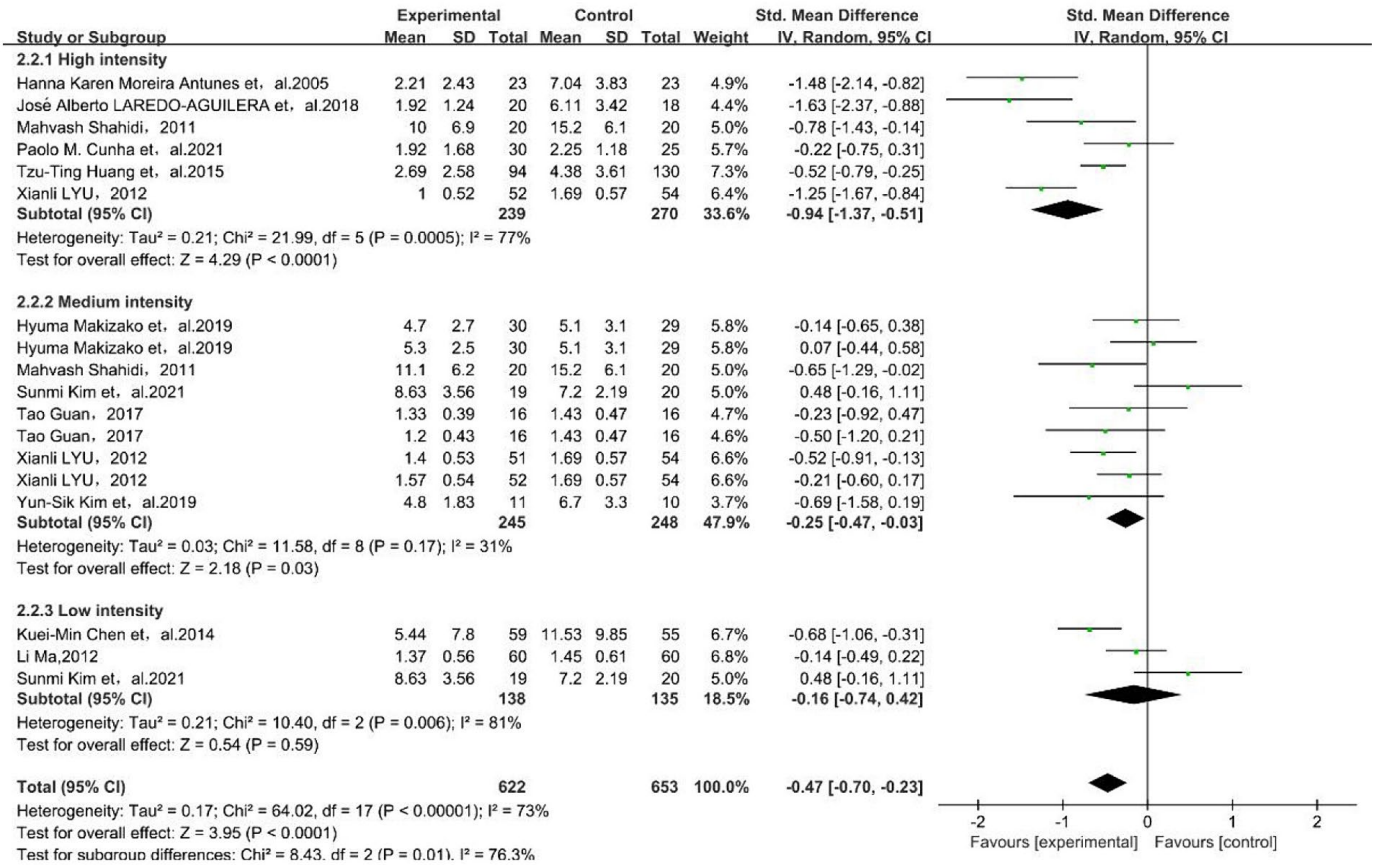
Forest plot for subgroup meta-analysis of physical exercise intensity. The green box and the black rhombus represent results of the individual studies and the combined results, respectively.

The results showed that medium physical exercise intensity [SMD = −0.25, 95% CI (−0.47, −0.03)] and high physical exercise intensity [SMD = −0.94, 95% CI (−1.37, −0.51)] had a better effect on depressive symptoms in older adults, as shown in Figure 4.

#### 3.3.3 Results of the exercise type subgroup analysis

**Figure 5:**
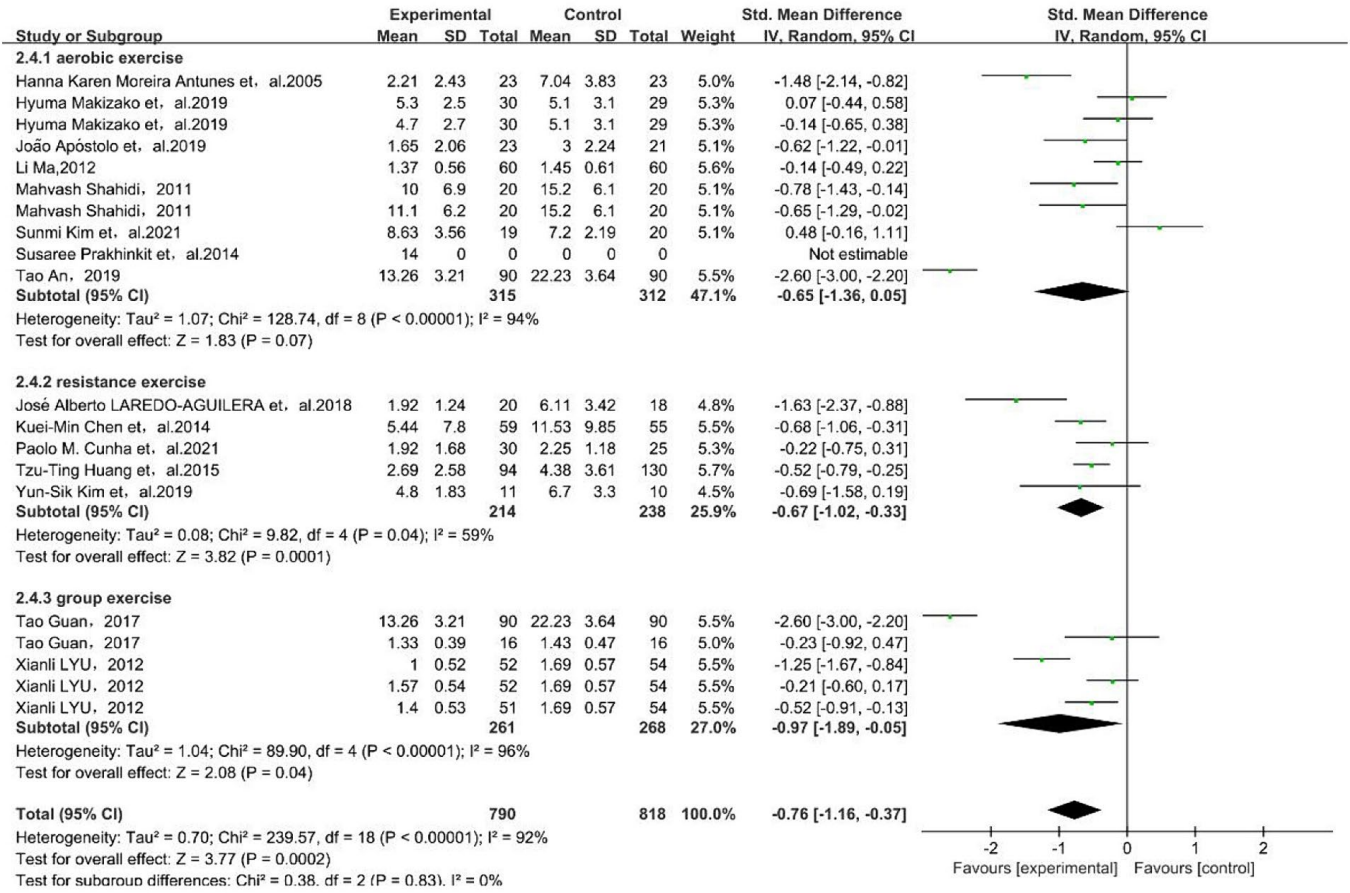
Forest plot for subgroup meta-analysis of exercise type. The green box and the black rhombus represent results of the individual studies and the combined results, respectively.

The results showed that resistance exercise [SMD = −0.70, 95% CI (−1.20, −0.20)] and group exercise [SMD = −0.97, 95% CI (−1.89, −0.05)] had a better effect on depressive symptoms in older adults, as shown in Figure 5.

### 3.4 Sensitivity analysis

The studies were sensitivity analyzed to identify heterogeneity sources, sequentially excluding the included individual studies one by one. The analysis results are shown in Supplementary Figure S3. One study (Lyu, 2012) may be the cause of the significant heterogeneity, and the results of the other studies did not change the combined results much, which means that the results of this study’s combined effect values are more stable.

### 3.5 Publication bias results

The 18 included studies were rubbed with Egger’s test for publication bias, respectively. The funnel plots were plotted with the effect values of individual studies as horizontal coordinates and the standard error of effect values as vertical coordinates (Supplementary Figure S4). The points of the included studies were arranged in a funnel shape, and Egger’s test (*p* = 0.731) showed no significant publication bias.

## 4 Discussion

This study used a meta-analysis to evaluate the impact of physical exercise on depressive symptoms in older adults. The findings showed that older adults who exercised significantly reduced their symptoms of depression. Specifically, moderate-intensity physical exercise, high-intensity physical exercise, resistance exercise, group exercise, and exercise with an intervention time of more than 3 months showed significant effectiveness in alleviating depressive symptoms in older adults. Notably, the results of the study showed that 6 months of exercise intervention time produced better results compared to 3 months of intervention time; resistance exercise showed a greater advantage than group exercise.

The results of this study suggest that both three-month and six-month exercise intervention times are highly effective in improving depressive symptoms in older adults. The number of studies have consistently shown that exercise intervention times lasting more than 12 weeks can significantly reduce depression levels in middle-aged and older individuals (Chen et al., 2023; Fang et al., 2015). In addition, the improvement effect of 6 months was superior to that of 3 months, and we can assume that there is a trend that longer exercise intervention time plays a key role in ensuring the positive effect of physical exercise on depressive symptoms.

The findings of this study provide evidence that engaging in moderate to high-intensity physical exercise can effectively improve depressive symptoms in older adults, aligning with the results of the current study (Mumba et al., 2021). Furthermore, the study demonstrates that both resistance exercise and group exercise were beneficial in alleviating depressive symptoms in older adults. Resistance exercise, specifically designed to enhance muscular strength and endurance, was found to be particularly effective. It is worth noting that resistance exercise has consistently demonstrated significant improvements in depressive symptoms in older individuals, consistent with the findings of the present study (Chen et al., 2023). It is worth noting that resistance exercise and moderate to high-intensity physical exercise promote the secretion of hormones such as dopamine, norepinephrine, and endorphins, which collectively contribute to mood enhancement and improved psychological well-being. Consequently, these physiological effects help alleviate depressive symptoms in older adults.

The decline in social relationships and physical functioning poses significant challenges for older adults with advancing age. However, it has been observed that taking part in social interactions can be helpful in reducing depressive symptoms in older adults (Lin, 2017). Social support, comprising both materialistic and emotional assistance from the community, family, relatives, or friends, plays a crucial role in this regard (Cullen, 1994). Exercise participation among older adults has been found to enhance peer support, thereby reducing the risk of depression (Yang et al., 2023). Moreover, research studies have underscored the importance of exercise as a means of social participation for older adults (Fang et al., 2015), with higher levels of social support correlating with decreased levels of depression (Hallgren et al., 2017). Furthermore, participation in group sports exercises offers valuable opportunities for social interaction among older adults.

This study did not find significant effects of aerobic exercise in improving depressive symptoms in older adults, which aligns with the findings of the study by Morres et al. (2019). The impact of yoga on depressive symptoms in older adults has yielded mixed results in previous research. Four studies demonstrated that yoga effectively improved depressive symptoms in older adults (Shapiro et al., 2007; Naveen et al., 2013; Kinser et al., 2014; Doria et al., 2015), while three studies reported no significant changes in depressive status. Interestingly, one study indicated that although yoga did not directly improve depression (Shapiro et al., 2007), it did prevent an intensification of depressive symptoms, which is consistent with the results of our study.

Although this study did not explore the interactive effects of lifestyle factors such as exercise, sleep, and nutrition on depression, it is important to acknowledge their collective influence in alleviating depressive symptoms (Chen et al., 2022a; Sejbuk et al., 2022; Chen et al., 2022c). Research shows that a combination of good sleep and regular exercise can significantly slow aging, reduce inflammation, enhance stress resilience (You et al., 2024b, 2024d, 2024e), and lower the risk of depression in older adults. However, poor sleep or low sleep quality can diminish the beneficial effects of exercise on depression, whereas regular exercise can improve sleep quality, creating a positive feedback loop (You et al., 2022, 2024a; You, 2024). Additionally, nutrition also plays a crucial role; healthy eating habits, such as the intake of active microorganisms, can enhance the effects of exercise and alleviate depressive symptoms by improving gut microbiota, reducing systemic inflammation, and regulating neurotransmitter levels (Chen et al., 2022b; You et al., 2024c, 2024f). Conversely, poor nutrition or an unbalanced diet can negate the positive effects of exercise on depression and may even exacerbate depressive symptoms. In conclusion, future studies should more comprehensively consider these potential confounding factors, especially the combined effects of lifestyle factors, and conduct more detailed analyses of gender differences and other socioeconomic factors to more accurately reveal the impact of different exercise interventions on depressive symptoms in older adults.

The findings of this systematic review have several potential clinical implications. First, the evidence suggests that structured exercise programs, particularly those focusing on aerobic and resistance training, can serve as an effective non-pharmacological strategy for managing depressive symptoms in older adults. This reinforces the importance of integrating physical activity into routine care for this population. Second, tailoring exercise interventions to the specific needs and capabilities of older adults may improve adherence and long-term mental health outcomes, offering a personalized approach to treatment. Finally, the incorporation of regular exercise into clinical practice could not only address depressive symptoms but also enhance physical health and overall quality of life, reducing the burden of comorbidities commonly associated with aging.

Some limitations of this study include the following, and future research could focus on these areas: (1) The number of included studies and sample size were small, and future studies could be added to the included studies; (2) The study searches are limited to English and Chinese, which may lead to linguistic or cultural bias; (3) The time of intervention used in the included studies was more concentrated at 3 and 6 months, and future studies could further refine the analysis of the sustained effect of different intervention times on the improved outcome. (4) This study did not analyze the impact of gender on depressive symptoms, which is a significant limitation. Previous research has shown that gender can influence depression outcomes (e.g., hormonal fluctuations in women). Future studies should include gender-specific analyses to better understand these differences. Additionally, expanding the sample size and diversity by including participants from various races, ethnicities, and geographic locations will help generalize findings across different subgroups of older adults. (5) This study did not account for socioeconomic factors such as household income, marital status, and chronic illness, future research should account for these variables to better understand their potential impact on depressive symptoms. (6) The use of different tools to measure depressive symptoms may affect the robustness of the meta-analysis. Although this study accounted for these variations, the inconsistencies in detecting and categorizing depressive symptoms across different tools could impact the comparability and interpretability of the results. Therefore, future research should aim for consistency in measurement tools to enhance the accuracy and comparability of findings. Adding these considerations would strengthen the validity of the findings and contribute to a more nuanced analysis of the intricate interplay between lifestyle factors, gender and socio-economic background that influence depressive symptoms in older adults. Additionally, future research should explore combining exercise with cognitive behavioral therapy (CBT) and mindfulness practices to potentially enhance depression management in older adults.

## 5 Conclusion

This study has demonstrated that engaging in physical exercise has a beneficial effect on the physical and emotional well-being of older adults, including preventing and alleviating depressive symptoms, while also contributing to the realization of sustainable health benefits. The effectiveness of exercise may be influenced by the specific way and intervention time. Participating in group programs can have positive psychological effects for older adults, as they take part in social interactions, relax, and receive emotional and social support, which helps alleviate depressive symptoms. Conversely, when engaging in more complex exercises like yoga, the expected exercise effects may not be fully achieved, resulting in minimal improvement. Ensuring appropriate exercise cycles is also crucial for optimizing the benefits of physical exercise.

To enhance the impact of physical exercise on depressive symptoms in older adults, several measures can be implemented. First, it is essential to guide and encourage more older adults to participate in physical exercise, aligning with the objectives of the Health China strategy. Second, promoting awareness and knowledge of physical health among older adults can help them identify suitable exercise options. Third, developing collaborative exercise programs for older adults can be beneficial. Moreover, providing and enhancing basic public sports facilities such as parks and community venues can improve accessibility and enjoyment of physical exercise for older adults. Finally, organizing collective exercises within the community can foster a sense of belonging and accomplishment among older adults, boosting their motivation to engage in physical exercise.By implementing these strategies, it is possible to create a supportive environment that encourages and facilitates physical exercise among older adults, ultimately leading to improved mental well-being and a reduction in depressive symptoms.

## Data Availability

The original contributions presented in the study are included in the article/Supplementary material, further inquiries can be directed to the corresponding authors.
