# Supplementary material for: Impact of exercise type, duration, and intensity on depressive symptoms in older adults: a systematic review and meta-analysis
Source: Front Psychol. 2024 Sep 13;15:1484172. doi: 10.3389/fpsyg.2024.1484172 (PMC11427357; doi:10.3389/fpsyg.2024.1484172)
Supplement: Supplementary file 1 [file Table_1.DOCX]

Supplementary Material

# Supplementary Tables

**Supplementary File**

Table S1. Search strategy for Cochrane Library, Web of Science, PubMed, Embase, CNKI, and Wanfang Data.

| Database | Number | Search strategy |
| --- | --- | --- |
| Pubmed | 584 | "aged"[MeSH Terms] OR "aged"[All Fields] OR ("older"[All Fields] AND "adults"[All Fields]) OR "older adults"[All Fields]AND (((((((((("Depression"[Mesh])) OR "Depressive Disorder"[Mesh]) OR (Depression, Postpartum[Title/Abstract])) OR (Depressive Disorder, Major[Title/Abstract])) OR (Depressive Disorder, Treatment-Resistant[Title/Abstract])) OR (Dysthymic Disorder[Title/Abstract])) OR (Premenstrual Dysphoric Disorder[Title/Abstract])) OR (Seasonal Affective Disorder[Title/Abstract])) OR (Vascular Depression[Title/Abstract]) AND (randomizedcontrolledtrial[Filter])) AND ((((((((((((Exercise[MeSH Terms]) OR (Cool-Down Exercise[Title/Abstract])) OR (Exergaming[Title/Abstract])) OR (Gymnastics[Title/Abstract])) OR (Muscle Stretching Exercises[Title/Abstract])) OR (Physical Conditioning, Animal[Title/Abstract])) OR (Physical Conditioning, Human[Title/Abstract])) OR (Preoperative Exercise[Title/Abstract])) OR (Running[Title/Abstract])) OR (Swimming[Title/Abstract])) OR (Walking[Title/Abstract])) OR (Warm-Up Exercise[Title/Abstract]) |
| Web of Science | 826 | exercise (TI) AND older adults (TI) AND depression (TI) NOT Preprint Citation Index |
| EMBASE | 181 | depression: ab,ti AND exercise:ab,ti AND older adults,ti AND ([controlled clinical trial]/lim OR [randomized controlled trial]/lim |
| Cochrane | 834 | depression in Title Abstract Keyword AND older adults in Title Abstract Keyword AND exercise in Title Abstract Keyword - (Word variations have been searched) |
| Wanfang | 91 | "exercise" AND "depression" AND "older adults" |
| CNKI | 72 | "exercise" AND "depression" AND "older adults" |

^S1^The search encompassed studies available until January 7, 2024.

Table S2. Literature excluded after reading the full text

| Elimination of causes（number） | Reference |
| --- | --- |
| Not RCT（21） | (Towle et al., 1989; Brenes et al., 2007; Baxter et al., 2010; Chalé-Rush et al., 2010; Midtgaard et al., 2011; Taspinar et al., 2014; Sawamoto et al., 2016; Hallgren et al., 2017; Rahman et al., 2018; Taani et al., 2018; Hernandez et al., 2019; Reid et al., 2019; Strid et al., 2019; Abdelbasset et al., 2020; Hansen et al., 2020; Boolani et al., 2021; Khoo et al., 2021; Salihu et al., 2021; Brush et al., 2022; Kim et al., 2023; Yu et al., 2023) |
| Inconsistent outcome indicators (27) | (Blumenthal et al., 1991; McNeil et al., 1991; McMurdo and Burnett, 1992; Partonen et al., 1998; Tsutsumi et al., 1998; Singh et al., 2001; Mather et al., 2002; Penninx et al., 2002; Craft et al., 2007; Milani and Lavie, 2009; Oeland et al., 2010; Callaghan et al., 2011; Guo et al., 2012; Riebe et al., 2012; Tekur et al., 2012; Pereira et al., 2013; Belvederi Murri et al., 2015; Chen et al., 2015; Doose et al., 2015; van Beljouw et al., 2015; Bang et al., 2016; Schuver and Lewis, 2016; Cothran et al., 2017; Noradechanunt et al., 2017; López-Torres Hidalgo and DEP-EXERCISE Group, 2019; Roh et al., 2020) |
| Not relevant to the theme (8) | (Schechtman et al., 1997; Singh et al., 1997; Mata et al., 2013; Newton et al., 2014; Abrahão et al., 2016; Lynch et al., 2017; Cartmel et al., 2021; Galvão et al., 2021) |
| Inconsistent participants （52） | (Martinsen et al., 1989; King et al., 1993; Palmer, 1995; Blumenthal et al., 1999; Babyak et al., 2000; Leppämäki et al., 2002; Wang et al., 2002, 2022; Atlantis et al., 2004; Bartholomew et al., 2005; Delahanty et al., 2006; Daley et al., 2007; Knubben et al., 2007; Neuberger et al., 2007; Hoffman et al., 2008; Kerr et al., 2008; Tomas-Carus et al., 2008; Williams and Tappen, 2008; Sims et al., 2009; Chan et al., 2012a, 2012b; Jensen et al., 2012; Xiong et al., 2012a, 2012b; Leung et al., 2013; Bernard et al., 2015; Schlenstedt et al., 2015; Gao et al., 2016; Kai et al., 2016; Uebelacker et al., 2017; Arrieta et al., 2018; Assunção Júnior et al., 2018; Wunram et al., 2018; Abdelbasset and Alqahtani, 2019; Averill et al., 2019; de Lima et al., 2019; Johnson et al., 2019; Kruisdijk et al., 2019; Kwok et al., 2019; Langoni et al., 2019; Lin et al., 2019; Suzuki et al., 2019; Danielsson et al., 2020; Haussleiter et al., 2020; Imboden et al., 2020; Gilbody et al., 2021; Huang et al., 2021; Kablan et al., 2022; Seo et al., 2023; Wagner et al., 2023; Zare et al., 2023; Fogawat et al., 2024) |
| Inconsistent intervention measures （18） | (Kugler et al., 1990; Damush et al., 2008; Pakkala et al., 2008; Payne et al., 2008; Franco et al., 2010; Penttinen et al., 2011; Piette et al., 2011; Brown et al., 2014; Jacquart et al., 2014; Ansai and Rebelatto, 2015; Kwon, 2015; Snyder et al., 2016; Strid et al., 2016; Lok et al., 2017; Soucy et al., 2017; Moderators of Response to Cognitive Behavior Therapy for Major Depression in Patients With Heart Failure: Erratum, 2019; Stahl et al., 2020; Hidalgo et al., 2021) |

# Reference

Abdelbasset, W. K., and Alqahtani, B. A. (2019). A randomized controlled trial on the impact of moderate-intensity continuous aerobic exercise on the depression status of middle-aged patients with congestive heart failure. *Medicine (Baltimore)* 98, e15344. doi: 10.1097/MD.0000000000015344

Abdelbasset, W. K., Alrawaili, S. M., Nambi, G., Yassen, E., Moawd, S. A., and Ahmed, A. S. (2020). Therapeutic effects of proprioceptive exercise on functional capacity, anxiety, and depression in patients with diabetic neuropathy: a 2-month prospective study. *Clin Rheumatol* 39, 3091–3097. doi: 10.1007/s10067-020-05086-4

Abrahão, M. I., Gomiero, A. B., Peccin, M. S., Grande, A. J., and Trevisani, V. F. M. (2016). Cardiovascular training vs. resistance training for improving quality of life and physical function in patients with systemic lupus erythematosus: a randomized controlled trial. *Scand J Rheumatol* 45, 197–201. doi: 10.3109/03009742.2015.1094126

Ansai, J. H., and Rebelatto, J. R. (2015). Effect of two physical exercise protocols on cognition and depressive symptoms in oldest-old people: A randomized controlled trial. *Geriatr Gerontol Int* 15, 1127–1134. doi: 10.1111/ggi.12411

Arrieta, H., Rezola-Pardo, C., Echeverria, I., Iturburu, M., Gil, S. M., Yanguas, J. J., et al. (2018). Physical activity and fitness are associated with verbal memory, quality of life and depression among nursing home residents: preliminary data of a randomized controlled trial. *BMC Geriatr* 18, 80. doi: 10.1186/s12877-018-0770-y

Assunção Júnior, J. C., de Almeida Silva, H. J., da Silva, J. F. C., da Silva Cruz, R., de Almeida Lins, C. A., and de Souza, M. C. (2018). Zumba dancing can improve the pain and functional capacity in women with fibromyalgia. *J Bodyw Mov Ther* 22, 455–459. doi: 10.1016/j.jbmt.2017.09.022

Atlantis, E., Chow, C.-M., Kirby, A., and Singh, M. F. (2004). An effective exercise-based intervention for improving mental health and quality of life measures: a randomized controlled trial. *Prev Med* 39, 424–434. doi: 10.1016/j.ypmed.2004.02.007

Averill, I. R. E., Beaglehole, B., Douglas, K. M., Jordan, J., Crowe, M. T., Inder, M., et al. (2019). Activation therapy for the treatment of inpatients with depression - protocol for a randomised control trial compared to treatment as usual. *BMC Psychiatry* 19, 52. doi: 10.1186/s12888-019-2038-2

Babyak, M., Blumenthal, J. A., Herman, S., Khatri, P., Doraiswamy, M., Moore, K., et al. (2000). Exercise treatment for major depression: maintenance of therapeutic benefit at 10 months. *Psychosom Med* 62, 633–638. doi: 10.1097/00006842-200009000-00006

Bang, K. S., Lee, I. S., Kim, S. J., Song, M. K., and Park, S. E. (2016). [The Effects of Urban Forest-walking Program on Health Promotion Behavior, Physical Health, Depression, and Quality of Life: A Randomized Controlled Trial of Office-workers]. *J Korean Acad Nurs* 46, 140–148. doi: 10.4040/jkan.2016.46.1.140

Bartholomew, J. B., Morrison, D., and Ciccolo, J. T. (2005). Effects of acute exercise on mood and well-being in patients with major depressive disorder. *Med Sci Sports Exerc* 37, 2032–2037. doi: 10.1249/01.mss.0000178101.78322.dd

Baxter, H., Winder, R., Chalder, M., Wright, C., Sherlock, S., Haase, A., et al. (2010). Physical activity as a treatment for depression: the TREAD randomised trial protocol. *Trials* 11, 105. doi: 10.1186/1745-6215-11-105

Belvederi Murri, M., Amore, M., Menchetti, M., Toni, G., Neviani, F., Cerri, M., et al. (2015). Physical exercise for late-life major depression. *Br J Psychiatry* 207, 235–242. doi: 10.1192/bjp.bp.114.150516

Bernard, P., Ninot, G., Bernard, P. L., Picot, M. C., Jaussent, A., Tallon, G., et al. (2015). Effects of a six-month walking intervention on depression in inactive post-menopausal women: a randomized controlled trial. *Aging Ment Health* 19, 485–492. doi: 10.1080/13607863.2014.948806

Blumenthal, J. A., Babyak, M. A., Moore, K. A., Craighead, W. E., Herman, S., Khatri, P., et al. (1999). Effects of exercise training on older patients with major depression. *Arch Intern Med* 159, 2349–2356. doi: 10.1001/archinte.159.19.2349

Blumenthal, J. A., Emery, C. F., Madden, D. J., Schniebolk, S., Walsh-Riddle, M., George, L. K., et al. (1991). Long-term effects of exercise on psychological functioning in older men and women. *J Gerontol* 46, P352-361. doi: 10.1093/geronj/46.6.p352

Boolani, A., Sur, S., Yang, D., Avolio, A., Goodwin, A., Mondal, S., et al. (2021). Six Minutes of Physical Activity Improves Mood in Older Adults: A Pilot Study. *J Geriatr Phys Ther* 44, 18–24. doi: 10.1519/JPT.0000000000000233

Brenes, G. A., Williamson, J. D., Messier, S. P., Rejeski, W. J., Pahor, M., Ip, E., et al. (2007). Treatment of minor depression in older adults: a pilot study comparing sertraline and exercise. *Aging Ment Health* 11, 61–68. doi: 10.1080/13607860600736372

Brown, R. A., Abrantes, A. M., Minami, H., Read, J. P., Marcus, B. H., Jakicic, J. M., et al. (2014). A preliminary, randomized trial of aerobic exercise for alcohol dependence. *J Subst Abuse Treat* 47, 1–9. doi: 10.1016/j.jsat.2014.02.004

Brush, C. J., Hajcak, G., Bocchine, A. J., Ude, A. A., Muniz, K. M., Foti, D., et al. (2022). A randomized trial of aerobic exercise for major depression: examining neural indicators of reward and cognitive control as predictors and treatment targets. *Psychol Med* 52, 893–903. doi: 10.1017/S0033291720002573

Callaghan, P., Khalil, E., Morres, I., and Carter, T. (2011). Pragmatic randomised controlled trial of preferred intensity exercise in women living with depression. *BMC Public Health* 11, 465. doi: 10.1186/1471-2458-11-465

Cartmel, B., Hughes, M., Ercolano, E. A., Gottlieb, L., Li, F., Zhou, Y., et al. (2021). Randomized trial of exercise on depressive symptomatology and brain derived neurotrophic factor (BDNF) in ovarian cancer survivors: The Women’s Activity and Lifestyle Study in Connecticut (WALC). *Gynecol Oncol* 161, 587–594. doi: 10.1016/j.ygyno.2021.02.036

Chalé-Rush, A., Guralnik, J. M., Walkup, M. P., Miller, M. E., Rejeski, W. J., Katula, J. A., et al. (2010). Relationship between physical functioning and physical activity in the lifestyle interventions and independence for elders pilot. *J Am Geriatr Soc* 58, 1918–1924. doi: 10.1111/j.1532-5415.2010.03008.x

Chan, W., Immink, M. A., and Hillier, S. (2012a). Yoga and exercise for symptoms of depression and anxiety in people with poststroke disability: a randomized, controlled pilot trial. *Altern Ther Health Med* 18, 34–43.

Chan, W., Immink, M. A., and Hillier, S. (2012b). Yoga and exercise for symptoms of depression and anxiety in people with poststroke disability: a randomized, controlled pilot trial. *Altern Ther Health Med* 18, 34–43.

Chen, H.-M., Tsai, C.-M., Wu, Y.-C., Lin, K.-C., and Lin, C.-C. (2015). Randomised controlled trial on the effectiveness of home-based walking exercise on anxiety, depression and cancer-related symptoms in patients with lung cancer. *Br J Cancer* 112, 438–445. doi: 10.1038/bjc.2014.612

Cothran, F. A., Paun, O., Barnes, L. L., Epps, F., Schoeny, M., and Farran, C. J. (2017). Comparing the Effect of a Moderate Physical Activity Intervention on the Mental Health Outcomes of African American and Caucasian Dementia Family Caregivers: A Secondary Data Analysis. *Issues Ment Health Nurs* 38, 996–1004. doi: 10.1080/01612840.2017.1364807

Craft, L. L., Freund, K. M., Culpepper, L., and Perna, F. M. (2007). Intervention study of exercise for depressive symptoms in women. *J Womens Health (Larchmt)* 16, 1499–1509. doi: 10.1089/jwh.2007.0483

Daley, A. J., Crank, H., Saxton, J. M., Mutrie, N., Coleman, R., and Roalfe, A. (2007). Randomized trial of exercise therapy in women treated for breast cancer. *J Clin Oncol* 25, 1713–1721. doi: 10.1200/JCO.2006.09.5083

Damush, T. M., Wu, J., Bair, M. J., Sutherland, J. M., and Kroenke, K. (2008). Self-management practices among primary care patients with musculoskeletal pain and depression. *J Behav Med* 31, 301–307. doi: 10.1007/s10865-008-9156-5

Danielsson, L., Waern, M., Hensing, G., and Holmgren, K. (2020). Work-directed rehabilitation or physical activity to support work ability and mental health in common mental disorders: a pilot randomized controlled trial. *Clin Rehabil* 34, 170–181. doi: 10.1177/0269215519880230

de Lima, T. A., Ferreira-Moraes, R., Alves, W. M. G. da C., Alves, T. G. G., Pimentel, C. P., Sousa, E. C., et al. (2019). Resistance training reduces depressive symptoms in elderly people with Parkinson disease: A controlled randomized study. *Scand J Med Sci Sports* 29, 1957–1967. doi: 10.1111/sms.13528

Delahanty, L. M., Conroy, M. B., Nathan, D. M., and Diabetes Prevention Program Research Group (2006). Psychological predictors of physical activity in the diabetes prevention program. *J Am Diet Assoc* 106, 698–705. doi: 10.1016/j.jada.2006.02.011

Doose, M., Ziegenbein, M., Hoos, O., Reim, D., Stengert, W., Hoffer, N., et al. (2015). Self-selected intensity exercise in the treatment of major depression: A pragmatic RCT. *Int J Psychiatry Clin Pract* 19, 266–275. doi: 10.3109/13651501.2015.1082599

Fogawat, K., Keswani, J., Sharma, H., Tewani, G. R., Kodali, P. B., and Nair, P. M. K. (2024). Randomized controlled trial investigating the role of yoga at workplace in improving fatigue, burnout, pain, strength, and quality of life among blue-collar workers. *J Educ Health Promot* 13, 152. doi: 10.4103/jehp.jehp_541_23

Franco, C., Mañas, I., Cangas, A. J., Moreno, E., and Gallego, J. (2010). Reducing teachers’ psychological distress through a mindfulness training program. *Span J Psychol* 13, 655–666. doi: 10.1017/s1138741600002328

Galvão, D. A., Newton, R. U., Chambers, S. K., Spry, N., Joseph, D., Gardiner, R. A., et al. (2021). Psychological distress in men with prostate cancer undertaking androgen deprivation therapy: modifying effects of exercise from a year-long randomized controlled trial. *Prostate Cancer Prostatic Dis* 24, 758–766. doi: 10.1038/s41391-021-00327-2

Gao, L., Zhang, L., Qi, H., and Petridis, L. (2016). Middle-aged Female Depression in Perimenopausal Period and Square Dance Intervention. *Psychiatr Danub* 28, 372–378.

Gilbody, S., Littlewood, E., McMillan, D., Chew-Graham, C. A., Bailey, D., Gascoyne, S., et al. (2021). Behavioural activation to prevent depression and loneliness among socially isolated older people with long-term conditions: The BASIL COVID-19 pilot randomised controlled trial. *PLoS Med* 18, e1003779. doi: 10.1371/journal.pmed.1003779

Guo, P., East, L., and Arthur, A. (2012). A preoperative education intervention to reduce anxiety and improve recovery among Chinese cardiac patients: a randomized controlled trial. *Int J Nurs Stud* 49, 129–137. doi: 10.1016/j.ijnurstu.2011.08.008

Hallgren, M., Lundin, A., Tee, F. Y., Burström, B., and Forsell, Y. (2017). Somebody to lean on: Social relationships predict post-treatment depression severity in adults. *Psychiatry Res* 249, 261–267. doi: 10.1016/j.psychres.2016.12.060

Hansen, H., Bieler, T., Beyer, N., Kallemose, T., Wilcke, J. T., Østergaard, L. M., et al. (2020). Supervised pulmonary tele-rehabilitation versus pulmonary rehabilitation in severe COPD: a randomised multicentre trial. *Thorax* 75, 413–421. doi: 10.1136/thoraxjnl-2019-214246

Haussleiter, I. S., Bolsinger, B., Assion, H.-J., and Juckel, G. (2020). Adjuvant Guided Exercise Therapy Versus Self-Organized Activity in Patients With Major Depression. *J Nerv Ment Dis* 208, 982–988. doi: 10.1097/NMD.0000000000001240

Hernandez, R., Andrade, F. C. D., Piedra, L. M., Tabb, K. M., Xu, S., and Sarkisian, C. (2019). The impact of exercise on depressive symptoms in older Hispanic/Latino adults: results from the “¡Caminemos!” study. *Aging Ment Health* 23, 680–685. doi: 10.1080/13607863.2018.1450833

Hidalgo, J. L.-T., Sotos, J. R., and DEP-EXERCISE Group (2021). Effectiveness of Physical Exercise in Older Adults With Mild to Moderate Depression. *Ann Fam Med* 19, 302–309. doi: 10.1370/afm.2670

Hoffman, B. M., Blumenthal, J. A., Babyak, M. A., Smith, P. J., Rogers, S. D., Doraiswamy, P. M., et al. (2008). Exercise fails to improve neurocognition in depressed middle-aged and older adults. *Med Sci Sports Exerc* 40, 1344–1352. doi: 10.1249/MSS.0b013e31816b877c

Huang, F., Leng, X., Kasukurthi, M. V., Huang, Y., Li, D., Tan, S., et al. (2021). Utilizing Machine Learning Techniques to Predict the Efficacy of Aerobic Exercise Intervention on Young Hypertensive Patients Based on Cardiopulmonary Exercise Testing. *J Healthc Eng* 2021, 6633832. doi: 10.1155/2021/6633832

Imboden, C., Gerber, M., Beck, J., Holsboer-Trachsler, E., Pühse, U., and Hatzinger, M. (2020). Aerobic exercise or stretching as add-on to inpatient treatment of depression: Similar antidepressant effects on depressive symptoms and larger effects on working memory for aerobic exercise alone. *J Affect Disord* 276, 866–876. doi: 10.1016/j.jad.2020.07.052

Jacquart, S. D., Marshak, H. H., Dos Santos, H., Luu, S. M., Berk, L. S., McMahon, P. T., et al. (2014). The effects of simultaneous exercise and psychotherapy on depressive symptoms in inpatient, psychiatric older adults. *Adv Mind Body Med* 28, 8–17.

Jensen, C. D., Aylward, B. S., and Steele, R. G. (2012). Predictors of attendance in a practical clinical trial of two pediatric weight management interventions. *Obesity (Silver Spring)* 20, 2250–2256. doi: 10.1038/oby.2012.96

Johnson, J. E., Stout, R. L., Miller, T. R., Zlotnick, C., Cerbo, L. A., Andrade, J. T., et al. (2019). Randomized cost-effectiveness trial of group interpersonal psychotherapy (IPT) for prisoners with major depression. *J Consult Clin Psychol* 87, 392–406. doi: 10.1037/ccp0000379

Kablan, N., Uzun, S., Kulalı, F., and Tatar, Y. (2022). Plantar Venous Pump Activity, Generalized Joint Hypermobility, and Foot Mobility in Ballerinas: A Case-Control Study. *J Sport Rehabil* 31, 582–588. doi: 10.1123/jsr.2021-0278

Kai, Y., Nagamatsu, T., Kitabatake, Y., and Sensui, H. (2016). Effects of stretching on menopausal and depressive symptoms in middle-aged women: a randomized controlled trial. *Menopause* 23, 827–832. doi: 10.1097/GME.0000000000000651

Kerr, J., Patrick, K., Norman, G., Stein, M. B., Calfas, K., Zabinski, M., et al. (2008). Randomized control trial of a behavioral intervention for overweight women: impact on depressive symptoms. *Depress Anxiety* 25, 555–558. doi: 10.1002/da.20320

Khoo, I., Close, J. C. T., Lord, S. R., Delbaere, K., and Taylor, M. E. (2021). Relationship between Depressive Symptoms and Cognitive, Psychological, and Physical Performance in Community-Dwelling Older People with Cognitive Impairment. *Dement Geriatr Cogn Disord* 50, 482–490. doi: 10.1159/000520853

Kim, H., Lee, K., Lee, Y. H., Park, Y., Park, Y., Yu, Y., et al. (2023). The Effectiveness of a Mobile Phone-Based Physical Activity Program for Treating Depression, Stress, Psychological Well-Being, and Quality of Life Among Adults: Quantitative Study. *JMIR Mhealth Uhealth* 11, e46286. doi: 10.2196/46286

King, A. C., Taylor, C. B., and Haskell, W. L. (1993). Effects of differing intensities and formats of 12 months of exercise training on psychological outcomes in older adults. *Health Psychol* 12, 292–300. doi: 10.1037//0278-6133.12.4.292

Knubben, K., Reischies, F. M., Adli, M., Schlattmann, P., Bauer, M., and Dimeo, F. (2007). A randomised, controlled study on the effects of a short-term endurance training programme in patients with major depression. *Br J Sports Med* 41, 29–33. doi: 10.1136/bjsm.2006.030130

Kruisdijk, F., Hopman-Rock, M., Beekman, A. T. F., and Hendriksen, I. (2019). EFFORT-D: results of a randomised controlled trial testing the EFFect of running therapy on depression. *BMC Psychiatry* 19, 170. doi: 10.1186/s12888-019-2156-x

Kugler, J., Dimsdale, J. E., Hartley, L. H., and Sherwood, J. (1990). Hospital supervised vs home exercise in cardiac rehabilitation: effects on aerobic fitness, anxiety, and depression. *Arch Phys Med Rehabil* 71, 322–325.

Kwok, J. Y. Y., Kwan, J. C. Y., Auyeung, M., Mok, V. C. T., Lau, C. K. Y., Choi, K. C., et al. (2019). Effects of Mindfulness Yoga vs Stretching and Resistance Training Exercises on Anxiety and Depression for People With Parkinson Disease: A Randomized Clinical Trial. *JAMA Neurol* 76, 755–763. doi: 10.1001/jamaneurol.2019.0534

Kwon, S. H. (2015). Wheel of Wellness Counseling in Community Dwelling, Korean Elders: A Randomized, Controlled Trial. *J Korean Acad Nurs* 45, 459–468. doi: 10.4040/jkan.2015.45.3.459

Langoni, C. da S., Resende, T. de L., Barcellos, A. B., Cecchele, B., da Rosa, J. N., Knob, M. S., et al. (2019). The effect of group exercises on balance, mobility, and depressive symptoms in older adults with mild cognitive impairment: a randomized controlled trial. *Clin Rehabil* 33, 439–449. doi: 10.1177/0269215518815218

Leppämäki, S. J., Partonen, T. T., Hurme, J., Haukka, J. K., and Lönnqvist, J. K. (2002). Randomized trial of the efficacy of bright-light exposure and aerobic exercise on depressive symptoms and serum lipids. *J Clin Psychiatry* 63, 316–321.

Leung, R. W. M., McKeough, Z. J., Peters, M. J., and Alison, J. A. (2013). Short-form Sun-style t’ai chi as an exercise training modality in people with COPD. *Eur Respir J* 41, 1051–1057. doi: 10.1183/09031936.00036912

Lin, F.-L., Yeh, M.-L., Lai, Y.-H., Lin, K.-C., Yu, C.-J., and Chang, J.-S. (2019). Two-month breathing-based walking improves anxiety, depression, dyspnoea and quality of life in chronic obstructive pulmonary disease: A randomised controlled study. *J Clin Nurs* 28, 3632–3640. doi: 10.1111/jocn.14960

Lok, N., Lok, S., and Canbaz, M. (2017). The effect of physical activity on depressive symptoms and quality of life among elderly nursing home residents: Randomized controlled trial. *Arch Gerontol Geriatr* 70, 92–98. doi: 10.1016/j.archger.2017.01.008

López-Torres Hidalgo, J. and DEP-EXERCISE Group (2019). Effectiveness of physical exercise in the treatment of depression in older adults as an alternative to antidepressant drugs in primary care. *BMC Psychiatry* 19, 21. doi: 10.1186/s12888-018-1982-6

Lynch, E., Mack, L. J., Karavolos, K., Avery, E., Liebman, R., Keim, K. S., et al. (2017). Recruitment and Baseline Characteristics of Participants in the Lifestyle Improvement Through Food and Exercise (LIFE) Study. *J Health Care Poor Underserved* 28, 463–486. doi: 10.1353/hpu.2017.0034

Martinsen, E. W., Hoffart, A., and Solberg, O. (1989). Comparing aerobic with nonaerobic forms of exercise in the treatment of clinical depression: a randomized trial. *Compr Psychiatry* 30, 324–331. doi: 10.1016/0010-440x(89)90057-6

Mata, J., Hogan, C. L., Joormann, J., Waugh, C. E., and Gotlib, I. H. (2013). Acute exercise attenuates negative affect following repeated sad mood inductions in persons who have recovered from depression. *J Abnorm Psychol* 122, 45–50. doi: 10.1037/a0029881

Mather, A. S., Rodriguez, C., Guthrie, M. F., McHarg, A. M., Reid, I. C., and McMurdo, M. E. T. (2002). Effects of exercise on depressive symptoms in older adults with poorly responsive depressive disorder: Randomised controlled trial. *The British Journal of Psychiatry* 180, 411–415. doi: 10.1192/bjp.180.5.411

McMurdo, M. E., and Burnett, L. (1992). Randomised controlled trial of exercise in the elderly. *Gerontology* 38, 292–298. doi: 10.1159/000213343

McNeil, J. K., LeBlanc, E. M., and Joyner, M. (1991). The effect of exercise on depressive symptoms in the moderately depressed elderly. *Psychol Aging* 6, 487–488. doi: 10.1037//0882-7974.6.3.487

Midtgaard, J., Stage, M., Møller, T., Andersen, C., Quist, M., Rørth, M., et al. (2011). Exercise may reduce depression but not anxiety in self-referred cancer patients undergoing chemotherapy. Post-hoc analysis of data from the “Body & Cancer” trial. *Acta Oncol* 50, 660–669. doi: 10.3109/0284186X.2010.543145

Milani, R. V., and Lavie, C. J. (2009). Impact of worksite wellness intervention on cardiac risk factors and one-year health care costs. *Am J Cardiol* 104, 1389–1392. doi: 10.1016/j.amjcard.2009.07.007

Moderators of Response to Cognitive Behavior Therapy for Major Depression in Patients With Heart Failure: Erratum (2019). *Psychosom Med* 81, 674. doi: 10.1097/PSY.0000000000000738

Neuberger, G. B., Aaronson, L. S., Gajewski, B., Embretson, S. E., Cagle, P. E., Loudon, J. K., et al. (2007). Predictors of exercise and effects of exercise on symptoms, function, aerobic fitness, and disease outcomes of rheumatoid arthritis. *Arthritis Rheum* 57, 943–952. doi: 10.1002/art.22903

Newton, K. M., Reed, S. D., Guthrie, K. A., Sherman, K. J., Booth-LaForce, C., Caan, B., et al. (2014). Efficacy of yoga for vasomotor symptoms: a randomized controlled trial. *Menopause* 21, 339–346. doi: 10.1097/GME.0b013e31829e4baa

Noradechanunt, C., Worsley, A., and Groeller, H. (2017). Thai Yoga improves physical function and well-being in older adults: A randomised controlled trial. *J Sci Med Sport* 20, 494–501. doi: 10.1016/j.jsams.2016.10.007

Oeland, A.-M., Laessoe, U., Olesen, A. V., and Munk-Jørgensen, P. (2010). Impact of exercise on patients with depression and anxiety. *Nord J Psychiatry* 64, 210–217. doi: 10.3109/08039480903511373

Pakkala, I., Read, S., Leinonen, R., Hirvensalo, M., Lintunen, T., and Rantanen, T. (2008). The effects of physical activity counseling on mood among 75- to 81-year-old people: a randomized controlled trial. *Prev Med* 46, 412–418. doi: 10.1016/j.ypmed.2007.11.002

Palmer, L. K. (1995). Effects of a walking program on attributional style, depression, and self-esteem in women. *Percept Mot Skills* 81, 891–898. doi: 10.2466/pms.1995.81.3.891

Partonen, T., Leppämäki, S., Hurme, J., and Lönnqvist, J. (1998). Randomized trial of physical exercise alone or combined with bright light on mood and health-related quality of life. *Psychol Med* 28, 1359–1364. doi: 10.1017/s0033291798007491

Payne, J. K., Held, J., Thorpe, J., and Shaw, H. (2008). Effect of exercise on biomarkers, fatigue, sleep disturbances, and depressive symptoms in older women with breast cancer receiving hormonal therapy. *Oncol Nurs Forum* 35, 635–642. doi: 10.1188/08.ONF.635-642

Penninx, B. W. J. H., Rejeski, W. J., Pandya, J., Miller, M. E., Di Bari, M., Applegate, W. B., et al. (2002). Exercise and depressive symptoms: a comparison of aerobic and resistance exercise effects on emotional and physical function in older persons with high and low depressive symptomatology. *J Gerontol B Psychol Sci Soc Sci* 57, P124-132. doi: 10.1093/geronb/57.2.p124

Penttinen, H. M., Saarto, T., Kellokumpu-Lehtinen, P., Blomqvist, C., Huovinen, R., Kautiainen, H., et al. (2011). Quality of life and physical performance and activity of breast cancer patients after adjuvant treatments. *Psychooncology* 20, 1211–1220. doi: 10.1002/pon.1837

Pereira, D. S., de Queiroz, B. Z., Miranda, A. S., Rocha, N. P., Felício, D. C., Mateo, E. C., et al. (2013). Effects of physical exercise on plasma levels of brain-derived neurotrophic factor and depressive symptoms in elderly women--a randomized clinical trial. *Arch Phys Med Rehabil* 94, 1443–1450. doi: 10.1016/j.apmr.2013.03.029

Piette, J. D., Richardson, C., Himle, J., Duffy, S., Torres, T., Vogel, M., et al. (2011). A randomized trial of telephonic counseling plus walking for depressed diabetes patients. *Med Care* 49, 641–648. doi: 10.1097/MLR.0b013e318215d0c9

Rahman, M. S., Forsell, Y., Hallgren, M., and Galanti, M. R. (2018). Tobacco use does not influence the response to non-pharmacologic depression treatment: A secondary analysis of the Regassa randomized controlled trial. *Psychiatry Res* 261, 442–448. doi: 10.1016/j.psychres.2018.01.006

Reid, K. F., Laussen, J., Bhatia, K., Englund, D. A., Kirn, D. R., Price, L. L., et al. (2019). Translating the Lifestyle Interventions and Independence for Elders Clinical Trial to Older Adults in a Real-World Community-Based Setting. *J Gerontol A Biol Sci Med Sci* 74, 924–928. doi: 10.1093/gerona/gly152

Riebe, G., Fan, M.-Y., Unützer, J., and Vannoy, S. (2012). Activity scheduling as a core component of effective care management for late-life depression. *Int J Geriatr Psychiatry* 27, 1298–1304. doi: 10.1002/gps.3784

Roh, H. W., Hong, C. H., Lim, H. K., Chang, K. J., Kim, H., Kim, N.-R., et al. (2020). A 12-week multidomain intervention for late-life depression: a community-based randomized controlled trial. *J Affect Disord* 263, 437–444. doi: 10.1016/j.jad.2019.12.013

Salihu, D., Wong, E. M. L., and Kwan, R. Y. C. (2021). Effects of an African Circle Dance Programme on Internally Displaced Persons with Depressive Symptoms: A Quasi-Experimental Study. *Int J Environ Res Public Health* 18, 843. doi: 10.3390/ijerph18020843

Sawamoto, R., Nozaki, T., Furukawa, T., Tanahashi, T., Morita, C., Hata, T., et al. (2016). Predictors of Dropout by Female Obese Patients Treated with a Group Cognitive Behavioral Therapy to Promote Weight Loss. *Obes Facts* 9, 29–38. doi: 10.1159/000442761

Schechtman, K. B., Kutner, N. G., Wallace, R. B., Buchner, D. M., and Ory, M. G. (1997). Gender, self-reported depressive symptoms, and sleep disturbance among older community-dwelling persons. FICSIT group. Frailty and Injuries: Cooperative Studies of Intervention Techniques. *J Psychosom Res* 43, 513–527. doi: 10.1016/s0022-3999(97)00117-7

Schlenstedt, C., Paschen, S., Kruse, A., Raethjen, J., Weisser, B., and Deuschl, G. (2015). Resistance versus Balance Training to Improve Postural Control in Parkinson’s Disease: A Randomized Rater Blinded Controlled Study. *PLoS One* 10, e0140584. doi: 10.1371/journal.pone.0140584

Schuver, K. J., and Lewis, B. A. (2016). Mindfulness-based yoga intervention for women with depression. *Complement Ther Med* 26, 85–91. doi: 10.1016/j.ctim.2016.03.003

Seo, E.-Y., Kim, Y.-S., Lee, Y.-J., and Hur, M.-H. (2023). Virtual Reality Exercise Program Effects on Body Mass Index, Depression, Exercise Fun and Exercise Immersion in Overweight Middle-Aged Women: A Randomized Controlled Trial. *Int J Environ Res Public Health* 20, 900. doi: 10.3390/ijerph20020900

Sims, J., Galea, M., Taylor, N., Dodd, K., Jespersen, S., Joubert, L., et al. (2009). Regenerate: assessing the feasibility of a strength-training program to enhance the physical and mental health of chronic post stroke patients with depression. *Int J Geriatr Psychiatry* 24, 76–83. doi: 10.1002/gps.2082

Singh, N. A., Clements, K. M., and Fiatarone, M. A. (1997). A randomized controlled trial of the effect of exercise on sleep. *Sleep* 20, 95–101. doi: 10.1093/sleep/20.2.95

Singh, N. A., Clements, K. M., and Singh, M. A. (2001). The efficacy of exercise as a long-term antidepressant in elderly subjects: a randomized, controlled trial. *J Gerontol A Biol Sci Med Sci* 56, M497-504. doi: 10.1093/gerona/56.8.m497

Snyder, P. J., Bhasin, S., Cunningham, G. R., Matsumoto, A. M., Stephens-Shields, A. J., Cauley, J. A., et al. (2016). Effects of Testosterone Treatment in Older Men. *N Engl J Med* 374, 611–624. doi: 10.1056/NEJMoa1506119

Soucy, I., Provencher, M., Fortier, M., and McFadden, T. (2017). Efficacy of guided self-help behavioural activation and physical activity for depression: a randomized controlled trial. *Cogn Behav Ther* 46, 493–506. doi: 10.1080/16506073.2017.1337806

Stahl, S. T., Smagula, S. F., Dew, M. A., Schulz, R., Albert, S. M., and Reynolds, C. F. (2020). Digital Monitoring of Sleep, Meals, and Physical Activity for Reducing Depression in Older Spousally-Bereaved Adults: A Pilot Randomized Controlled Trial. *Am J Geriatr Psychiatry* 28, 1102–1106. doi: 10.1016/j.jagp.2020.02.013

Strid, C., Andersson, C., Forsell, Y., Öjehagen, A., and Lundh, L.-G. (2016). Internet-based cognitive behaviour therapy and physical exercise - Effects studied by automated telephone assessments in mental ill-health patients; a randomized controlled trial. *Br J Clin Psychol* 55, 414–428. doi: 10.1111/bjc.12111

Strid, C., Hallgren, M., Forsell, Y., Kraepelien, M., and Öjehagen, A. (2019). Changes in alcohol consumption after treatment for depression: a secondary analysis of the Swedish randomised controlled study REGASSA. *BMJ Open* 9, e028236. doi: 10.1136/bmjopen-2018-028236

Suzuki, T., Kojima, N., Osuka, Y., Tokui, Y., Takasugi, S., Kawashima, A., et al. (2019). The Effects of Mold-Fermented Cheese on Brain-Derived Neurotrophic Factor in Community-Dwelling Older Japanese Women With Mild Cognitive Impairment: A Randomized, Controlled, Crossover Trial. *J Am Med Dir Assoc* 20, 1509-1514.e2. doi: 10.1016/j.jamda.2019.06.023

Taani, M. H., Siglinsky, E., Kovach, C. R., and Buehring, B. (2018). Psychosocial Factors Associated With Reduced Muscle Mass, Strength, and Function in Residential Care Apartment Complex Residents. *Res Gerontol Nurs* 11, 238–248. doi: 10.3928/19404921-20180810-02

Taspinar, B., Aslan, U. B., Agbuga, B., and Taspinar, F. (2014). A comparison of the effects of hatha yoga and resistance exercise on mental health and well-being in sedentary adults: a pilot study. *Complement Ther Med* 22, 433–440. doi: 10.1016/j.ctim.2014.03.007

Tekur, P., Nagarathna, R., Chametcha, S., Hankey, A., and Nagendra, H. R. (2012). A comprehensive yoga programs improves pain, anxiety and depression in chronic low back pain patients more than exercise: an RCT. *Complement Ther Med* 20, 107–118. doi: 10.1016/j.ctim.2011.12.009

Tomas-Carus, P., Gusi, N., Häkkinen, A., Häkkinen, K., Leal, A., and Ortega-Alonso, A. (2008). Eight months of physical training in warm water improves physical and mental health in women with fibromyalgia: a randomized controlled trial. *J Rehabil Med* 40, 248–252. doi: 10.2340/16501977-0168

Towle, D., Lincoln, N. B., and Mayfield, L. M. (1989). Service provision and functional independence in depressed stroke patients and the effect of social work intervention on these. *J Neurol Neurosurg Psychiatry* 52, 519–522. doi: 10.1136/jnnp.52.4.519

Tsutsumi, T., Don, B. M., Zaichkowsky, L. D., Takenaka, K., Oka, K., and Ohno, T. (1998). Comparison of high and moderate intensity of strength training on mood and anxiety in older adults. *Percept Mot Skills* 87, 1003–1011. doi: 10.2466/pms.1998.87.3.1003

Uebelacker, L. A., Kraines, M., Broughton, M. K., Tremont, G., Gillette, L. T., Epstein-Lubow, G., et al. (2017). Perceptions of hatha yoga amongst persistently depressed individuals enrolled in a trial of yoga for depression. *Complement Ther Med* 34, 149–155. doi: 10.1016/j.ctim.2017.06.008

van Beljouw, I. M. J., van Exel, E., van de Ven, P. M., Joling, K. J., Dhondt, T. D. F., Stek, M. L., et al. (2015). Does an outreaching stepped care program reduce depressive symptoms in community-dwelling older adults? A randomized implementation trial. *Am J Geriatr Psychiatry* 23, 807–817. doi: 10.1016/j.jagp.2014.09.012

Wagner, J. A., Bermúdez-Millán, A., Buckley, T. E., Buxton, O. M., Feinn, R. S., Kong, S., et al. (2023). Secondary analysis of a randomized trial testing community health educator interventions for diabetes prevention among refugees with depression: effects on nutrition, physical activity and sleep. *Int J Behav Nutr Phys Act* 20, 107. doi: 10.1186/s12966-023-01509-y

Wang, L., van Belle, G., Kukull, W. B., and Larson, E. B. (2002). Predictors of functional change: a longitudinal study of nondemented people aged 65 and older. *J Am Geriatr Soc* 50, 1525–1534. doi: 10.1046/j.1532-5415.2002.50408.x

Wang, Y., Luo, B., Wu, X., Li, X., and Liao, S. (2022). Comparison of the effects of Tai Chi and general aerobic exercise on weight, blood pressure and glycemic control among older persons with depressive symptoms: a randomized trial. *BMC Geriatr* 22, 401. doi: 10.1186/s12877-022-03084-6

Williams, C. L., and Tappen, R. M. (2008). Exercise training for depressed older adults with Alzheimer’s disease. *Aging Ment Health* 12, 72–80. doi: 10.1080/13607860701529932

Wunram, H. L., Hamacher, S., Hellmich, M., Volk, M., Jänicke, F., Reinhard, F., et al. (2018). Whole body vibration added to treatment as usual is effective in adolescents with depression: a partly randomized, three-armed clinical trial in inpatients. *Eur Child Adolesc Psychiatry* 27, 645–662. doi: 10.1007/s00787-017-1071-2

Xiong, G. L., Fiuzat, M., Kuchibhatla, M., Krishnan, R., O’Connor, C. M., Jiang, W., et al. (2012a). Health status and depression remission in patients with chronic heart failure: patient-reported outcomes from the SADHART-CHF trial. *Circ Heart Fail* 5, 688–692. doi: 10.1161/CIRCHEARTFAILURE.112.967620

Xiong, G. L., Fiuzat, M., Kuchibhatla, M., Krishnan, R., O’Connor, C. M., Jiang, W., et al. (2012b). Health status and depression remission in patients with chronic heart failure: patient-reported outcomes from the SADHART-CHF trial. *Circ Heart Fail* 5, 688–692. doi: 10.1161/CIRCHEARTFAILURE.112.967620

Yu, D. J., Yu, A. P., Leung, C. K., Chin, E. C., Fong, D. Y., Cheng, C. P., et al. (2023). Comparison of moderate and vigorous walking exercise on reducing depression in middle-aged and older adults: A pilot randomized controlled trial. *Eur J Sport Sci* 23, 1018–1027. doi: 10.1080/17461391.2022.2079424

Zare, H., Ibe, C. A., Yang, M., Porter, G., Gaston, M., Jones, N., et al. (2023). Evaluating the Impact of the Prime Time Sister Circles® Intervention on Reducing Depressive Symptoms Among African American Women with Uncontrolled Hypertension. *J Gen Intern Med* 38, 2879–2887. doi: 10.1007/s11606-023-08288-z

# Supplementary Figures


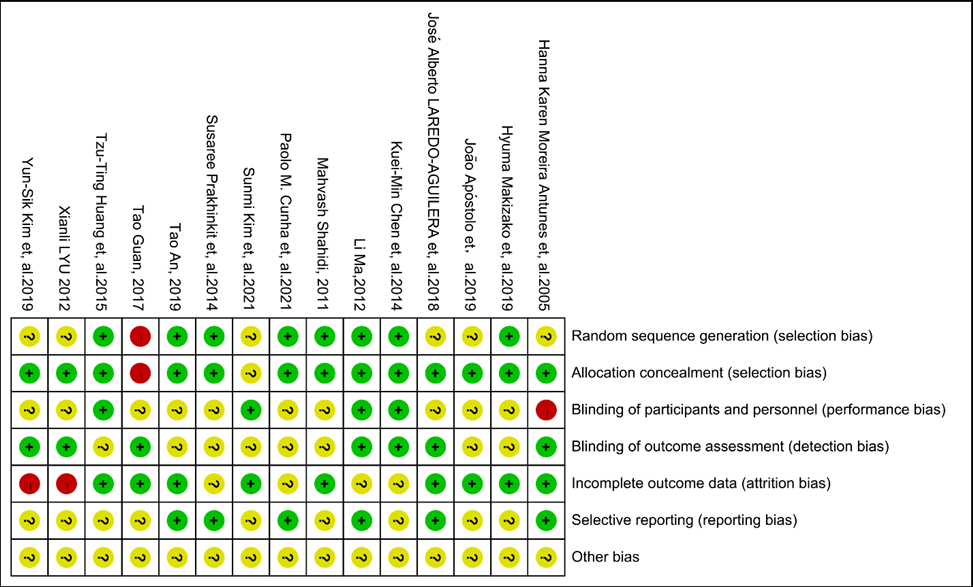


**Figure S1.** Risk of bias assessment of included studies.(+ : meet the criteria, − : not meet the criteria, ? : not clear).


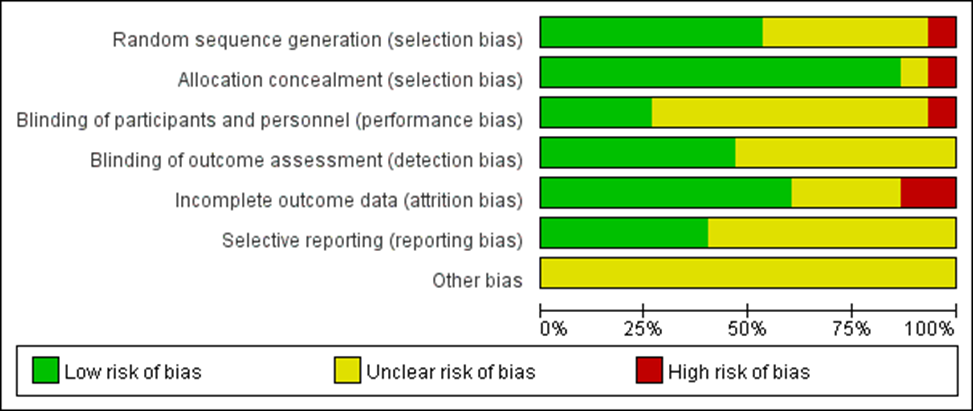


**Figure S2.** The overall risk of bias assessment of included studies.

**Figure S3**: Sensitivity analysis for meta-analysis of physical exercise on depressive symptoms in older adults. The vertical line in the middle represents the total combined effect size, and the two vertical lines on either side indicate the 95% confidence interval range for the total effect size. The point corresponding to each study indicates the combined effect size of the remaining studies after removing that study.

**Figure S4:** Funnel plot for meta-analysis of physical exercise on depressive symptoms in older adults. The vertical line in the middle represents the total combined effect size, and the two dashed lines on either side indicate the 95% confidence interval range for the total effect size. Each point corresponds to each study.
